# Attenuation of short-term autonomic cardiovascular responses with advancing gestational age

**DOI:** 10.3389/fphys.2026.1841598

**Published:** 2026-07-29

**Authors:** Manfred G. Moertl, Federica Piani, Helmut K. Lackner, Miha Lučovnik, Ivan Žebeljan, Adrian Mahlmann, Dejan Dinevski

**Affiliations:** 1 Faculty of Medicine, University of Maribor, Maribor, Slovenia; 2 Division of Physiology, Otto Loewi Research Center for Vascular Biology, Immunology and Inflammation, Medical University of Graz, Graz, Austria; 3 Sex and Gender in Disease Research Unit, Medical University of Graz, Graz, Austria; 4 Department of Perinatology, Division of Obstetrics and Gynaecology, University Medical Centre Ljubljana, Ljubljana, Slovenia; 5 Medical Faculty, University of Ljubljana, Ljubljana, Slovenia; 6 Department for Women’s Health, Health Center Lenart, Lenart v Slovenskih Goricah, Slovenia; 7 Vascular Center South Westphalia Hagen, Clinic of Angiology, St.-Josefs Hospital, Katholisches Krankenhaus Hagen, Hagen, Germany; 8 Department of Internal Medicine, University Hospital, Ruhr University Bochum, Bochum, Germany; 9 Medical Faculty, University of Maribor, Maribor, Slovenia

**Keywords:** autonomic nervous system, autonomic reactivity, cardio-respiratory coupling, heart rate variability, pregnancy

## Abstract

**Introduction:**

Pregnancy induces profound cardiovascular adaptations, including changes in vascular resistance, cardiac output, and heart rate, which are partly regulated by the autonomic nervous system. Although a shift toward reduced parasympathetic activity has been reported, the longitudinal evolution of autonomic function and its responsiveness to physiological stressors across gestation remain incompletely defined. This study aimed to characterize trimester-specific changes in autonomic regulation and reactivity.

**Methods:**

In this prospective longitudinal cohort, 67 healthy pregnant women were evaluated during the first (T1), second (T2), and third (T3) trimesters. Continuous cardiovascular monitoring was performed during a standardized protocol including rest, paced breathing, and a cognitive task. Heart rate variability, blood pressure, and phase synchronization indices were analysed using two-way repeated-measures ANOVA (Time × Phase).

**Results:**

Heart rate increased across gestation, while blood pressure showed a mid-pregnancy nadir. Indices of vagal modulation declined from T1 to T3, whereas overall variability remained unchanged. No effect of gestational age was observed for sympathovagal balance, but significant Time × Phase interactions indicated attenuation of autonomic reactivity. Both vagal responses to paced breathing and sympathetic responses to cognitive stress were reduced in late pregnancy. Similarly, indices of cardio-respiratory and cardiovascular coupling showed phase-dependent changes, with reduced synchronization during paced breathing in T3.

**Discussion:**

Our findings suggest that autonomic regulation during pregnancy is characterized by preserved responsiveness but reduced modulation. The decline in vagal activity, together with attenuated reactivity and reduced cardio-respiratory coupling, suggests reduced autonomic flexibility. These findings outline autonomic trajectories in healthy pregnancy that may serve as a normative reference against which early deviations could be examined in future studies on pregnancy-associated cardiovascular complications.

## 1 Introduction

Pregnancy functions as a natural physiological stress test for the female organism. The hemodynamic, metabolic, endocrine, and immunological demands imposed by gestation unmask the limits of regulatory systems and reveal otherwise latent susceptibilities to dysfunction (Duvekot and Peeters, 1994). This makes pregnancy a uniquely informative model for studying human physiology and pathophysiology. In addition, elucidating these mechanisms provides a powerful framework for understanding hemodynamic maladaptation in cardiometabolic diseases of pregnancy, which are rising sharply worldwide and pose a major global health challenge. At the core of hemodynamic regulation in pregnancy lies a tightly coordinated cardiovascular remodeling: circulating blood volume expands, systemic vascular resistance declines due to peripheral vasodilation and vascular remodeling, and heart rate and cardiac output rise to sustain both the maternal circulation and the increasing metabolic demands of the growing fetus (Duvekot and Peeters, 1994; Mahendru et al., 2014). These cardiac and vascular adaptations are finely regulated by the autonomic nervous system (ANS) (Ekholm, 1995; Fu and Levine, 2009; Brooks, 2023). The two primary branches of the ANS, the sympathetic and parasympathetic nervous systems, maintain a delicate balance that is crucial for cardiovascular homeostasis. Pregnancy is reported to be typically associated with a progressive increase in sympathetic activity and a relative reduction in parasympathetic tone, especially in the third trimester (Ekholm, 1995; Fu and Levine, 2009; Brooks, 2023). However, much of the current understanding stems from cross-sectional studies and comprehensive longitudinal evaluations across all three trimesters remain limited. Additionally, examining the autonomic response to physiological stimuli such as paced brathing or mental stress tasks may provide deeper insight into the adaptive flexibility of the maternal ANS during pregnancy (Weissman and Mendes, 2021). Understanding how ANS function evolves throughout pregnancy is essential for identifying normal physiological patterns and recognizing early signs of dysfunction that may provide a normative reference against which future studies could test whether deviations are associated with adverse outcomes such as preeclampsia, fetal growth restriction, or gestational diabetes (Lackner et al., 2019; Reyes et al., 2020).

Heart rate variability (HRV) provides a non-invasive and pregnancy-compatible method to probe autonomic regulation, offering time- and frequency-domain indices that reflect parasympathetic activity, sympathetic influences, and their dynamic balance (Lombardi, 2002). However, HRV at rest captures only a fraction of autonomic function, as baseline conditions may mask regulatory deficits or limit interpretability in the context of gestational heart rate acceleration. These limitations can be partially overcome by assessing autonomic responses to controlled physiological triggers, such as paced breathing or cognitive challenge which reveal modulation patterns and reactivity profiles that are not apparent under resting conditions. In addition, measures of cardiorespiratory synchronization offer complementary, indirect insight into autonomic coordination of cardiac and respiratory rhythms (Moertl et al., 2013; Ren and Zhang, 2019; Žebeljan et al., 2022).

In this prospective cohort study, we investigated trimester-specific changes in ANS regulation during healthy pregnancy. Heart rate variability (HRV) metrics and cardio-respiratory synchronization indices were assessed at rest, during paced breathing, and during a standardized cognitive challenge to probe parasympathetic activity, sympathetic influences, and their dynamic balance. Our aim was to characterize longitudinal trajectories of ANS function and to determine how autonomic reactivity to physiological and cognitive stressors evolves across gestation.

## 2 Methods

This longitudinal prospective cohort study used repeated measures across the first (T1: 12^+1^–16^+0^ weeks), second (T2: 21^+1^–25^+0^ weeks), and third (T3: 31^+0^–35^+0^ weeks) trimesters. The study adhered to the STROBE guidelines for observational research, with standardized afternoon measurement sessions and consistent instrumentation across visits.

### 2.1 Study population

The enrollment of participants in this prospective cohort study started in August 2020 and included seventy-four healthy pregnant women. Owing to the stringent artifact rejection criteria required for the calculation of phase-synchronization indices, which depend on simultaneous high-quality electrocardiography (ECG), continuous blood pressure, and impedance cardiography recordings, six women were excluded from the final analyses. Data were not replaced or extrapolated because artifacts affected multiple signal segments and involved either the first or the third measurement. After re-evaluation of the full dataset, one participant with an artifact limited to a single ECG channel was retained, resulting in a final analytical sample of 67 women. Exclusion criteria were: multiple pregnancies, the presence of one or more cardiovascular risk factors, cardiac arrhythmias, use of medications affecting heart rate or blood pressure, psychiatric disorders, epilepsy, kidney or liver disease, known fetal anomalies, autoimmune disorders, diabetes mellitus, and alcohol or illicit drug abuse. None of the participants developed hypertension or hypertensive disorders of pregnancy during the study, and office blood pressure remained below 140/90 mmHg at all three visits. All participants provided written informed consent. The study was approved by the Institutional Review Board of the University Medical Center Maribor (Pahor, 2022) and the Slovenian National Medical Ethics Committee (Project number 0120-575/2018/5; approved on 22 February 2019).

### 2.2 Measurement protocol

**Figure 1:**
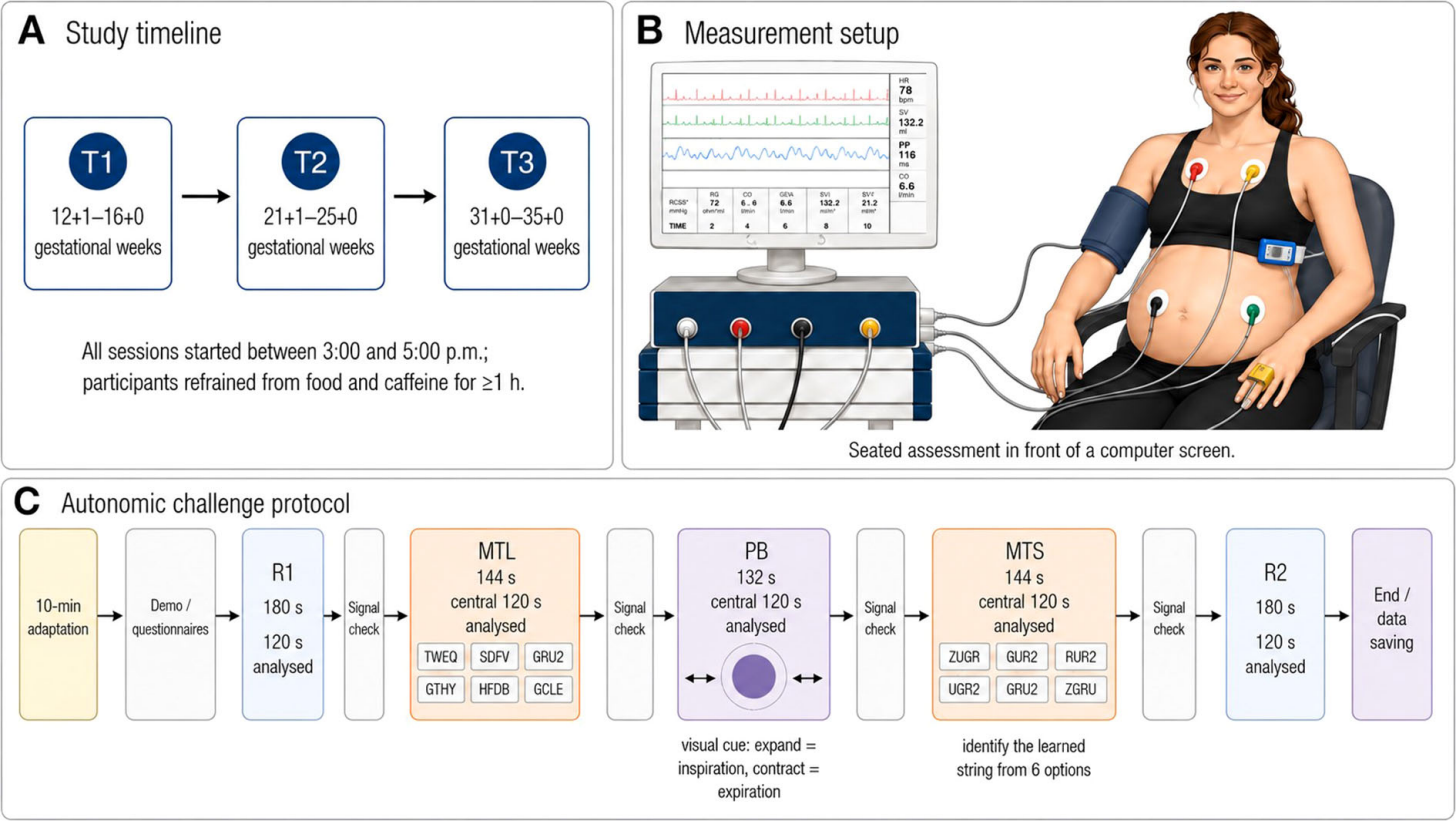
Study design, measurement setup, and autonomic challenge protocol. **(A)** Study timeline. Each participant was assessed at three time points: T1 (12 + 1–16 + 0 weeks), T2 (21 + 1–25 + 0 weeks), and T3 (31 + 0–35 + 0 gestational weeks). All sessions started between 3:00 and 5:00 p.m., and participants refrained from food and caffeine for at least one hour beforehand. **(B)** Measurement setup. Continuous cardiovascular monitoring was performed with the participant comfortably seated in front of a computer screen, using three-lead ECG, oscillometric brachial calibration with finger continuous blood pressure, and thoracic impedance. **(C)** Autonomic challenge protocol. After a 10-minute seated adaptation period and a task demonstration with questionnaires, the five analysed phases were performed sequentially, each separated by a brief signal-quality check: Rest 1 (R1, 180 s), Memory Task–Learning (MTL, 144 s), Paced Breathing (PB, 132 s), Memory Task–Selection (MTS, 144 s), and Rest 2 (R2, 180 s); the central 120 s of each phase were used for analysis. During PB, breathing was guided by a visual cue (a circle expanding on inspiration and contracting on expiration) at 10 breaths·min^-1^. During MTS, participants identified the previously learned four-letter string from six options. The total protocol lasted no longer than 30 minutes. The graphical illustration of the study protocol and measurement setup was generated and edited with the assistance of OpenAI ChatGPT image-generation tools. The final figure was curated by the authors to reflect the actual experimental setup and protocol.

Measurements were performed at three time points during pregnancy: T1, between 12^+1^ and 16^+0^ gestational weeks; T2, between 21^+1^ and 25^+0^ gestational weeks; and T3, between 31^+0^ and 35^+0^ gestational weeks. All measurements were performed in the afternoon, at approximately the same time of day between 3:00 and 5:00 p.m. For each participant, the three study visits were scheduled within this same time window across gestation, in order to minimize the potential influence of circadian variation on cardiovascular and autonomic parameters. Following familiarization with the test protocol, equipment, and study personnel, electrodes were attached, and participants were instructed to refrain from talking or making abrupt movements during the recordings. The study was performed in a quiet, temperature-controlled room located on the ground floor of a medical practice. Data acquisition was controlled by dedicated software, while the study investigator monitored signal quality and study progress on a separate screen. The participant station and the investigator’s station were visually separated by a room divider. To minimize interference with participants’ usual daily routines, no extensive restrictions were imposed before the measurements. This approach was considered appropriate given the repeated-measures design, in which each participant served as her own control. Nevertheless, participants were asked to avoid food and caffeine intake for at least one hour before the measurements. During the assessment, participants were seated in front of a computer screen and connected to the cardiovascular monitoring system, as shown in **Figure 1**.

Each session followed a standardized autonomic challenge protocol. After instrumentation and positioning, participants underwent a 10-minute seated adaptation period to allow cardiovascular and autonomic stabilization. Signal quality was checked before each active task condition, namely the memory task–learning phase (MTL), the paced-breathing condition, corresponding to the respiratory sinus arrhythmia (RSA) assessment, and the memory task–selection phase (MTS). The protocol started with a first resting recording period (R1), lasting 180 seconds, of which 120 seconds were used for analysis. The analysed segment corresponded to seconds 46–165 of the recording. R1 was followed by the memory task–learning phase (MTL). Before R1, participants had completed a short demonstration of the learning task; between the demonstration and R1, they filled in questionnaires unrelated to the present research question, including the Satisfaction With Life Scale. During MTL, participants were asked to memorize 12 four-letter character strings displayed on the screen in two rows of six strings. Each string was highlighted for 3 seconds, and the complete sequence was repeated four times, resulting in a total task duration of 144 seconds. The central 120 seconds of the MTL phase were used for analysis. The MTL phase was followed by the RSA assessment. Before the actual recording, participants completed a short demonstration to ensure that they were able to follow the visual breathing cue. During the task, a circle displayed on the screen expanded during inspiration and decreased in diameter during expiration, with a brief physiological pause between respiratory phases. The paced-breathing condition lasted 132 seconds, corresponding to 22 respiratory cycles of 6 seconds each, equivalent to 10 breaths per minute. The central 120 seconds were analysed. After paced breathing, a short signal check was performed to ensure high signal quality. Participants then completed the MTS phase, during which they were asked to identify the previously learned four-letter string from six response options, consisting of one correct target and five distractors. Responses had to be provided within a maximum time window of 144 seconds, and 120 seconds from the central part of the task were used for analysis. The protocol ended with a second resting recording period (R2), again lasting 180 seconds, with 120 seconds included in the analysis. The protocol was designed to induce autonomic modulation in both sympathetic and parasympathetic directions. The memory learning and selection tasks were intended to elicit cognitive load and sympathetic activation, whereas the paced-breathing/RSA condition was included to enhance vagal modulation. In addition, the paced-breathing phase served as a standardized delay period between learning and recall, allowing assessment of the subsequent physiological and cognitive response to the memory challenge.

### 2.3 Physiological data acquisition

Continuous monitoring was performed using the Task Force^®^ Monitor (CNSystems Medizintechnik AG, Graz, Austria) (Papousek et al., 2011), including:

- Blood Pressure: Sampled at 100 Hz (range: 50–250 mmHg; ± 5 mmHg), using a vascular unloading technique at the finger, corrected by oscillometric calibration on the contralateral upper arm.
- Heart Rate: R-R intervals derived from 3-lead ECG (sampling rate: 1 kHz; frequency band: 0.08–150 Hz).
- Thoracic Impedance: Sampled at 50 Hz (Z_0_ range: 10–75 Ω).

All raw data were exported to MATLAB^®^ (The MathWorks, Inc., Natick, MA, USA) for further analysis.

### 2.4 Signal processing and artifact correction

Artifact correction and interbeat interval calculation were performed using a semi-automatic software developed by our group (Lackner et al., 2019). Inter-beat interval series were processed with a semi-automatic artifact-handling software developed by our group and applied consistently across our studies. After artifact detection, signal stationarity was verified using Parseval’s theorem. Segments were retained only when valid data exceeded 95% for HRV analysis and 90% for continuous blood pressure and thoracic impedance. The 120-second analysis windows were selected to satisfy stationarity requirements (≥ 10× the wavelength of the lowest frequency of interest). The proportion of valid heart-rate data exceeded 99% in all phases and at all three time points, with no significant effect of Time, Phase, or their interaction (all p > 0.10; **Supplementary Table 1**), confirming comparable signal quality across gestation and protocol phases.

ECG artifacts were identified and corrected based on:

1. QRS complex pattern and timing to detect ectopic beats.
2. Individualized, age-dependent physiological limits.
3. Maximum change percentage relative to signal standard deviation.

The algorithm preserves full ECG information and ensures consistency. Continuous blood pressure values were subsequently verified using corrected ECG data and physiological constraints.

Physiological limits and signal change thresholds were calculated individually using the envelope of R-R intervals, systolic, and diastolic BP via Hilbert transformation. After artifact correction, beat-to-beat time series were resampled at 4 Hz using piecewise cubic spline interpolation to create equidistant time steps, facilitating frequency-domain and synchronization analyses.

### 2.5 Data segmentation and variable calculation

For all analyses, a stable 120-second resting interval following the adaptation phase was selected, ensuring signal stationarity (minimum duration being ~10× the wavelength of the lowest frequency of interest). Heart rate variability (HRV) metrics were computed in accordance with established guidelines (Task Force of the European Society of Cardiology and the North American Society of Pacing and Electrophysiology, 1996). Time-domain measures included:

- SDNN: Standard deviation of R-R intervals.
- RMSSD: Root mean square of successive differences between adjacent R-R intervals.

Frequency-domain analysis employed Burg’s method (model order = 24) after detrending with a second-order polynomial. Frequency bands were defined as:

- Low Frequency (LF): 0.04–0.15 Hz
- High Frequency (HF): 0.15-0.40 Hz

Due to skewed distributions, natural logarithmic transformation was applied to frequency variables, with LF/HF presented as ln(LF/HF).

Blood Pressure: Mean values were computed for systolic, diastolic and mean arterial pressure using the recording of the continuous blood pressure signal.

Respiratory Frequency (RF): Derived from thoracic impedance signals, band-pass filtered (4th-order Butterworth filter; 0.05–0.8 Hz), then downsampled to 4 Hz. Respiratory frequency (RF) was calculated as the mean breathing rate using a pattern recognition method to detect individual breathing cycles.

### 2.6 Cardio-respiratory phase synchronization

For the calculation of phase synchronization variables among the R–R interval, systolic blood pressure (SBP), and respiratory signals, we employed custom software developed by our research group (Moertl et al., 2013; Lackner et al., 2019).

Phase synchronization analysis was used to assess the dynamic interactions between cardiac, respiratory, and vascular oscillators. This approach is particularly suitable because these physiological systems behave as weakly coupled oscillators, whose phase relationships are sensitive to external perturbations such as cognitive or emotional challenges (e.g., the memory task) and to controlled physiological inputs (e.g., paced breathing). Thus, changes in synchronization patterns can reveal how autonomic regulation adapts to both stress-related activation and vagal modulation (Bartsch et al., 2012).

In this study, three synchronization indices were computed:

1. γ_SBP * RR_ synchronization index between systolic blood pressure and RR interval (inter-beat interval between all successive heartbeats)
2. γ_RF * RR_ synchronization index between respiratory frequency and RR interval
3. γ_RF * SBP_ synchronization index between respiratory frequency and systolic blood pressure

### 2.7 Statistical analysis

Statistical analyses were performed using IBM SPSS Statistics for Windows, Version 26.0 (IBM Corp., Armonk, NY, USA). A two-way repeated-measures ANOVA was used to assess the effects of gestational age and experimental phase on all outcome variables, with Time (T1, T2, T3) and Phase (R1, MTL, PB, MTS, R2) as within-subject factors. Analyses were performed on complete cases.

For each outcome, the main effects of Time and Phase, as well as the Time × Phase interaction, were tested. Results are reported as mean ± standard deviation, F-statistics, degrees of freedom, p-values, and partial eta squared (ηp²). When the assumption of sphericity was violated, Greenhouse–Geisser-corrected degrees of freedom and p-values were reported. Partial eta squared values were interpreted cautiously using conventional benchmarks, with ηp² values of approximately 0.01, 0.06, and 0.14 considered indicative of small, medium, and large effects, respectively. Effects with ηp² ≥ 0.06 were considered potentially relevant, whereas effects approaching or exceeding ηp² = 0.14 were regarded as being of particular interest.

When significant main effects were observed, Bonferroni-adjusted *post hoc* pairwise comparisons were performed on estimated marginal means. In the presence of a significant Time × Phase interaction, simple-effects analyses were conducted, followed by Bonferroni-adjusted pairwise comparisons. A two-tailed p-value ≤ 0.05 was considered statistically significant.

## 3 Results

A total of 74 women were recruited. Seven were excluded due to artifact handling, leaving 67 women of European ancestry. Maternal age was 29.9 ± 4.0 years [range 22–38] and 44 (66%) were nulliparous. Mean BMI increased from 23.8 ± 3.3 kg/m^2^ (T1) to 25.3 ± 3.5 kg/m^2^ (T2) and 27.0 ± 3.7 kg/m^2^ (T3).

### 3.1 Paced breathing and memory task

Mean breathing frequencies during the software-controlled paced breathing were 9.8 ± 1.0 min^-1^ (T1), 9.6 ± 0.3 min^-1^ (T2) and 9.9 ± 1.3 min^-1^ (T3), *F*(2, 132) = 1.9, p = 0.16. The average number of correct responses during the memory task did not differ significantly across the three measurements (T1: 7.7 ± 1.8, T2: 8.1 ± 1.6, T3: 7.5 ± 2.6 out of 12; F(2, 132) = 3.0, p = 0.053). However, the processing time increased significantly (*F*(2, 132) = 7.8, p < 0.001). The *post-hoc* test (Bonferroni corrected) revealed that the processing time during the third trimester measurement (T3) (6.4 ± 1.0 s) was significantly higher compared to measurements T1 (5.9 ± 1.1 s) and T2 (6.0 ± 0.9 s).

### 3.2 Heart rate and heart rate variability

Heart rate and HRV were analysed in both the time domain (heart rate, SDNN, RMSSD; **Table 1**) and the frequency domain (ln(LF), ln(HF), ln(LF/HF); **Table 2**). The repeated-measures ANOVA revealed significant effects of Time, Phase, and their interaction (Time × Phase) on heart rate, indicating that heart rate differed significantly both across measurement sessions and between the phases of the experimental protocol. *Post-hoc* analyses (Bonferroni-corrected) showed a significantly higher heart rate at T3 compared to T1 and T2 during resting period 1, paced breathing, and the solving phase of the memory task. No significant main effect of Time was found for SDNN. The vagal-related parameter RMSSD showed significantly lower values at T3 compared to T1 and T2 during resting period 1 and paced breathing. The significant Time × Phase interaction for all time domain parameters was further supported by supplementary analyses on delta values (change relative to R1; F(4.56, 301.04) = 10.6, p <.001, ηp^2^ = .14).

**Table 1:** Heart rate and time domain heart rate variability parameters.

	R1	MTL	PB	MTS	R2	*F-statistics*
Heart rate (bpm)						
T1^3^	83.4 ± 10.2	93.1 ± 12.5	88.1 ± 9.9	89.2 ± 11.0	82.6 ± 10.0	*Time*: F(2, 132) = 15.0; **p <.001**; η_p_^2^ = .19 *Phase*: F(2.54, 167.75) = 54.3; **p <.001**; η_p_^2^ = .45 *Time × Phase*: F(5.98, 394.67) = 14.0; **p <.001**; η_p_^2^ = .18
T2^3^	84.7 ± 9.4	89.7 ± 11.4	87.3 ± 8.5	88.2 ± 9.9	82.8 ± 8.9	
T3^1, 2^	90.4 ± 10.3	93.3 ± 10.3	92.0 ± 9.5	92.1 ± 9.1	88.7 ± 9.3	
SDNN = standard deviation of inter-beat intervals (ms)						
T1	38.0 ± 13.2	33.3 ± 10.4	52.7 ± 16.5	41.0 ± 13.1	39.3 ± 13.4	*Time*: F(1.79, 118.23) = 0.5; p = .572; η_p_^2^ = .01 *Phase*: F(3.38, 222.96) = 46.2; **p <.001**; η_p_^2^ = .41 *Time × Phase*: F(8, 528) = 3.9; **p <.001**; η_p_^2^ = .06
T2	37.6 ± 13.4	33.6 ± 10.7	49.4 ± 15.0	36.9 ± 12.1	40.1 ± 13.7	
T3	39.5 ± 15.7	36.1 ± 14.3	47.7 ± 15.3	37.3 ± 12.0	39.7 ± 14.3	
RMSSD = root mean square of successive R–R interval differences (ms)						
T1^3^	30.2 ± 14.3	22.8 ± 11.5	34.9 ± 13.9	23.0 ± 8.3	30.6 ± 14.6	*Time*: F(1.79, 118.17) = 8.4; **p <.001**; η_p_^2^ = .11 *Phase*: F(3.09, 204.10) = 33.6; **p <.001**; η_p_^2^ = .34 *Time × Phase*: F(5.43, 358.15) = 6.1; **p <.001**; η_p_^2^ = .08
T2^3^	28.3 ± 14.5	24.6 ± 13.6	31.9 ± 13.7	22.7 ± 11.2	30.3 ± 13.7	
T3^1, 2^	23.7 ± 14.0	21.5 ± 12.6	25.6 ± 12.6	20.8 ± 11.5	24.5 ± 13.2	

Data are mean ± SD (n = 67). T1, 12^+1^–16^+0^ weeks; T2, 21^+1^–25^+0^ weeks; T3, 31^+0^–35^+0^ weeks of gestation. F-statistics from a 3 (Time) × 5 (Phase) repeated-measures ANOVA; Greenhouse–Geisser-corrected degrees of freedom reported where Mauchly’s test p <.05. Bold p-values: p <.05. Time superscripts: ^1^differs from T1; ^2^differs from T2; ^3^differs from T3 (Bonferroni-corrected). R1 (a), Rest period 1; MTL (b), Memory Task (learning); PB (c), Paced Breathing; MTS (d), Memory Task (solving); R2 (e), Rest period 2. Phase *post-hoc* (Bonferroni, all p <.001 unless noted): HR, all pairs significant except c vs d (ns). SDNN, all pairs significant except a vs d, a vs e, c vs d, c vs e (ns). RMSSD, all pairs significant except c vs d, a vs e (ns).

**Table 2:** Frequency domain heart rate variability parameters.

	R1	MTL	PB	MTS	R2	*F-statistics*
ln(LF) = natural logarithm of low-frequency power (ms²)						
T1^3^	5.84 ± 0.85	5.50 ± 0.85	6.02 ± 0.68	6.40 ± 0.78	5.97 ± 0.85	*Time*: F(2, 132) = 28.1; **p <.001**; η_p_^2^ = .30 *Phase*: F(3.34, 220.45) = 22.4; **p <.001**; η_p_^2^ = .25 *Time × Phase*: F(8, 528) = 2.4; **p <.05**; η_p_^2^ = .03
T2^3^	5.69 ± 0.84	5.52 ± 0.70	6.08 ± 0.74	6.02 ± 0.86	5.86 ± 0.93	
T3^1, 2^	5.35 ± 0.85	5.23 ± 0.71	5.74 ± 0.73	5.79 ± 0.85	5.48 ± 0.73	
ln(HF) = natural logarithm of high-frequency power (ms²)						
T1^3^	5.82 ± 0.97	5.20 ± 0.94	7.05 ± 0.85	5.34 ± 0.76	5.86 ± 0.99	*Time*: F(2, 132) = 9.5; **p <.001**; η_p_^2^ = .13 *Phase*: F(3.19, 210.70) = 156.9; **p <.001**; η_p_^2^ = .70 *Time × Phase*: F(8, 528) = 5.2; **p <.001**; η_p_^2^ = .07
T2^3^	5.71 ± 1.02	5.28 ± 0.99	6.74 ± 0.90	5.26 ± 0.93	5.89 ± 0.94	
T3^1, 2^	5.34 ± 1.13	5.07 ± 1.08	6.33 ± 0.91	5.13 ± 1.00	5.51 ± 1.05	
ln(LF/HF) = natural logarithm of the low- to high-frequency power ratio						
T1	0.02 ± 0.91	0.30 ± 0.82	−1.02 ± 0.85	1.05 ± 0.68	0.11 ± 0.90	*Time*: F(2, 132) = 0.2; p = .804; η_p_^2^ = .00 *Phase*: F(3.50, 230.87) = 104.6; **p <.001**; η_p_^2^ = .61 *Time × Phase*: F(8, 528) = 5.1; **p <.001**; η_p_^2^ = .07
T2	−0.01 ± 0.99	0.24 ± 0.93	−0.66 ± 0.92	0.76 ± 0.92	−0.03 ± 0.97	
T3	0.01 ± 0.99	0.16 ± 1.02	−0.59 ± 0.92	0.66 ± 0.82	−0.02 ± 0.86	

Data are mean ± SD (n = 67). T1, 12^+1^–16^+0^ weeks; T2, 21^+1^–25^+0^ weeks; T3, 31^+0^–35^+0^ weeks of gestation. F-statistics from a 3 (Time) × 5 (Phase) repeated-measures ANOVA; Greenhouse–Geisser-corrected degrees of freedom reported where Mauchly’s test p <.05. Bold p-values: p <.05. Time superscripts: ^1^differs from T1; ^2^differs from T2; ^3^differs from T3 (Bonferroni-corrected). R1 (a), Rest period 1; MTL (b), Memory Task (learning); PB (c), Paced Breathing; MTS (d), Memory Task (solving); R2 (e), Rest period 2. Phase *post-hoc* (Bonferroni, all p <.001 unless noted,) ln(LF), all pairs significant except a vs e, b vs e (ns). ln(HF), all pairs significant except b vs d (ns). ln(LF/HF), all pairs significant except a vs. e (ns).

**Figure 2:**
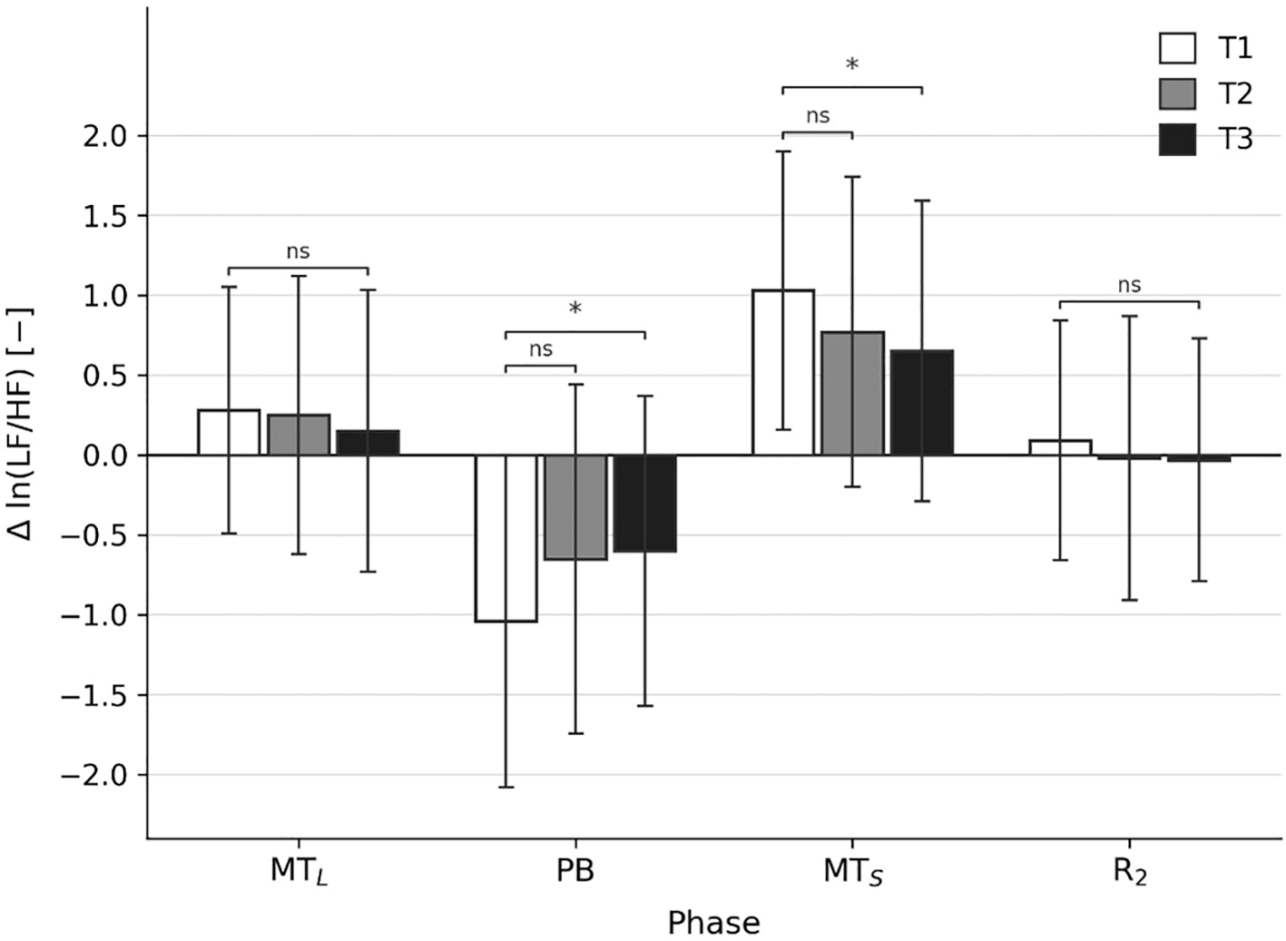
Changes in the sympathovagal balance (Δln[LF/HF]) relative to rest across protocol phases and gestational time points. Δln(LF/HF) represents the change in the natural logarithm of the low- to high-frequency power ratio relative to Rest period 1 (R1). Bars show mean ± SD at T1 (12^+1^–16^+0^ weeks), T2 (21^+1^–25^+0^ weeks), and T3 (31^+0^–35^+0^ weeks). Phase *post-hoc* comparisons (Bonferroni-corrected, all p <.001): at all three time points, all phases differed significantly from each other. Brackets indicate pairwise comparisons between time points within each phase; *p <.05; ns, not significant.

In the frequency domain, both ln(LF) and ln(HF) showed significant main effects of Time, Phase, and their interaction (**Table 2**). Both spectral components were significantly lower at T3 compared to T1 and T2, with no significant differences between T1 and T2. The ln(LF/HF) ratio showed no significant main effect of Time, but a significant Phase effect and Time × Phase interaction were observed. As shown in **Figure 2**, delta values relative to R1 revealed higher vagally mediated values during paced breathing at T1 and T2 compared to T3, and higher sympathetically driven values during the memory task at T1 and T2 compared to T3. The significant interaction was confirmed by supplementary analyses on delta values (F(4.56, 301.04) = 10.6, p <.001, ηp^2^ = .14).

### 3.3 Blood pressure variables

The results of the repeated-measures ANOVA for blood pressure variables are presented in **Table 3**. Supplementary analyses showed that systolic blood pressure (SBP) was significantly lower at T2 compared to T1 and T3 during the resting phases, a pattern that was similarly reflected in mean arterial pressure (MAP) and diastolic blood pressure (DBP). No significant effects of paced breathing or memory task performance were observed for SBP, whereas MAP and DBP remained significantly lower at T2 relative to T1 and T3. Supplementary analyses on delta values confirmed that the Time × Phase interaction was not significant for any blood pressure variable (SBP: p = .07; MAP: p = .106; DBP: p = .125).

**Table 3:** Blood pressure variables.

	R1	MTL	PB	MTS	R2	*F-statistics*
SBP (mmHg)						
T1^2^	108.6 ± 9.8	113.4 ± 11.4	109.0 ± 11.2	113.1 ± 13.4	108.1 ± 10.6	*Time*: F(2, 132) = 8.7; **p <.001**; η_p_^2^ = .12 *Phase*: F(3.06, 202.03) = 22.4; **p <.001**; ηp^2^ = .25 *Time × Phase*: F(6.46, 426.01) = 1.8; p = .092; η_p_^2^ = .03
T2^1, 3^	105.6 ± 10.1	108.9 ± 10.4	105.1 ± 10.2	109.7 ± 12.2	107.1 ± 9.8	
T3^2^	109.5 ± 9.4	112.8 ± 10.2	107.2 ± 9.1	112.6 ± 12.2	109.9 ± 8.5	
MAP (mmHg)						
T1^2^	85.0 ± 8.0	89.1 ± 8.9	84.6 ± 9.1	89.4 ± 10.2	84.7 ± 8.3	*Time*: F(2, 132) = 13.0; **p <.001**; η_p_^2^ = .16 *Phase*: F(3.16, 208.38) = 36.2; **p <.001**; η_p_^2^ = .35 *Time × Phase*: F(6.31, 416.42) = 1.6; p = .147; η_p_^2^ = .02
T2^1, 3^	82.5 ± 8.4	85.4 ± 8.9	81.0 ± 8.6	85.7 ± 9.8	82.9 ± 7.9	
T3^2^	85.9 ± 7.5	88.5 ± 7.9	83.6 ± 7.2	89.3 ± 9.2	86.5 ± 7.1	
DBP (mmHg)						
T1^2^	68.8 ± 7.2	72.8 ± 7.9	68.3 ± 8.0	73.1 ± 8.2	68.7 ± 6.9	*Time*: F(2, 132) = 16.4; **p <.001**; η_p_^2^ = .20 *Phase*: F(3.19, 210.75) = 38.9; **p <.001**; η_p_^2^ = .37 *Time × Phase*: F(6.12, 403.71) = 1.5; p = .168; η_p_^2^ = .02
T2^1, 3^	66.7 ± 7.8	69.6 ± 8.4	65.2 ± 8.4	69.6 ± 9.0	66.5 ± 6.9	
T3^2^	70.2 ± 6.5	72.5 ± 6.9	68.3 ± 6.5	73.8 ± 7.6	71.0 ± 6.3	

Data are mean ± SD (n = 67). T1, 12^+1^–16^+0^ weeks; T2, 21^+1^–25^+0^ weeks; T3, 31^+0^–35^+0^ weeks of gestation. F-statistics from a 3 (Time) × 5 (Phase) repeated-measures ANOVA; Greenhouse–Geisser-corrected degrees of freedom reported where Mauchly’s test p <.05. Bold p-values: p <.05. Time superscripts: ^1^differs from T1; ^2^differs from T2; ^3^differs from T3 (Bonferroni-corrected). R1 (a), Rest period 1; MTL (b), Memory Task (learning); PB (c), Paced Breathing; MTS (d), Memory Task (solving); R2 (e), Rest period 2. Phase *post-hoc* (Bonferroni, all p <.001 unless noted), SBP, all pairs significant except a vs c, a vs e, c vs e (ns). MAP and DBP, all pairs significant except a vs e (ns).

### 3.4 Phase synchronization indices

Phase synchronization analysis was used to assess the dynamic interactions between cardiac, respiratory, and vascular oscillators. This approach is particularly suitable because these physiological systems behave as weakly coupled oscillators, whose phase relationships are sensitive to external perturbations such as cognitive challenges (e.g., the memory task) and controlled physiological inputs (e.g., paced breathing). Three synchronization indices were computed: γSBP×RR (synchronization index between systolic blood pressure and RR-interval), γRR×Resp (synchronization index between RR-interval and respiratory frequency), and γSBP×Resp (synchronization index between systolic blood pressure and respiratory frequency). Results are presented in **Table 4**.

**Table 4:** Phase synchronization indices.

	R1	MTL	PB	MTS	R2	*F-statistics*
γSBP × RR (−)						
T1	0.66 ± 0.22	0.56 ± 0.23	0.84 ± 0.18	0.43 ± 0.21	0.67 ± 0.20	*Time*: F(2, 132) = 0.4; p = .674; η_p_^2^ = .01 *Phase*: F(3.12, 206.24) = 61.9; **p <.001**; η_p_^2^ = .48 *Time × Phase*: F(8, 528) = 5.1; **p <.001**; η_p_^2^ = .07
T2	0.62 ± 0.22	0.55 ± 0.23	0.76 ± 0.24	0.49 ± 0.22	0.64 ± 0.23	
T3	0.62 ± 0.20	0.58 ± 0.23	0.73 ± 0.25	0.51 ± 0.22	0.65 ± 0.21	
γRR × Resp (−)						
T1	0.69 ± 0.21	0.59 ± 0.26	0.84 ± 0.18	0.40 ± 0.21	0.67 ± 0.22	*Time*: F(1.76, 116.08) = 1.6; p = .202; ηp^2^ = .02 *Phase*: F(3.54, 233.67) = 94.2; **p <.001**; ηp^2^ = .59 *Time × Phase*: F(8, 528) = 4.4; **p <.001**; η_p_^2^ = .06
T2	0.71 ± 0.20	0.63 ± 0.23	0.86 ± 0.13	0.47 ± 0.23	0.70 ± 0.21	
T3	0.67 ± 0.24	0.64 ± 0.24	0.80 ± 0.20	0.53 ± 0.22	0.71 ± 0.20	
γSBP × Resp (−)						
T1	0.72 ± 0.23	0.65 ± 0.24	0.78 ± 0.23	0.43 ± 0.24	0.73 ± 0.21	*Time*: F(2, 132) = 0.6; p = .544; η_p_^2^ = .01 *Phase*: F(3.52, 232.11) = 41.9; **p <.001**; η_p_^2^ = .39 *Time × Phase*: F(8, 528) = 6.4; **p <.001**; η_p_^2^ = .09
T2	0.68 ± 0.22	0.61 ± 0.25	0.75 ± 0.22	0.48 ± 0.26	0.66 ± 0.24	
T3	0.67 ± 0.23	0.67 ± 0.24	0.68 ± 0.27	0.55 ± 0.24	0.70 ± 0.22	

Data are mean ± SD (n = 67). T1, 12^+1^–16^+0^ weeks; T2, 21^+1^–25^+0^ weeks; T3, 31^+0^–35^+0^ weeks of gestation. F-statistics from a 3 (Time) × 5 (Phase) repeated-measures ANOVA; Greenhouse–Geisser-corrected degrees of freedom reported where Mauchly’s test p <.05. Bold p-values: p <.05. Time superscripts: ^1^differs from T1; ^2^differs from T2; ^3^differs from T3 (Bonferroni-corrected). R1 (a), Rest period 1; MTL (b), Memory Task (learning); PB (c), Paced Breathing; MTS (d), Memory Task (solving); R2 (e), Rest period 2. Phase *post-hoc* (Bonferroni, all p <.001), γSBP×RR and γRR×Resp, all pairs significant except a vs e (ns). γSBP×Resp, all pairs significant except a vs b, a vs c, b vs c, b vs e, c vs e (ns).

No significant main effect of Time was observed for any of the three synchronization indices. However, the Time × Phase interaction was significant for all three variables (delta-value analyses: all p <.001), indicating that the temporal pattern of coupling across the experimental phases differed between gestational stages. Consistent with the behaviour of weakly coupled oscillators, coupling decreased significantly during paced breathing with advancing gestation, while no significant changes were observed under resting conditions. Significant differences between T1 and T3 were also found during the solving phase of the memory task.

## 4 Discussion

This prospective longitudinal study examined how autonomic control evolves across pregnancy by analysing HRV metrics, cardiovascular dynamics, and cardio-respiratory phase coupling. To elicit distinct autonomic responses, the protocol alternated between resting conditions, paced breathing, and a standardized cognitive challenge. This design allowed us to probe parasympathetic activation, sympathetic engagement, and subsequent recovery, providing an integrated view of autonomic adaptability across gestation. To our knowledge, this is the first study to provide a comprehensive, multi-phase assessment of autonomic regulation across all trimesters of healthy pregnancy, integrating HRV, beat-to-beat cardiovascular measures, and cardio-respiratory phase coupling within a standardized challenge protocol.

Evidence on autonomic regulation in pregnancy suggests a shift in baseline tone across gestation, with reports of reduced vagal indices and signs of relative sympathetic predominance in later trimesters (Speranza et al., 1998; Brooks, 2023). Two longitudinal studies are particularly relevant for situating our contribution. Garg et al. (2020) assessed HRV, blood pressure variability, and cardiac baroreflex sensitivity in healthy pregnant women at gestational windows comparable to ours (11–13, 20–22, and 30–32 weeks), using lead-II ECG and beat-to-beat blood pressure recorded at rest in the supine position. They reported a progressive decline in overall HRV and baroreflex sensitivity, together with an increase in blood pressure variability and a rising LF/HF ratio across gestation, interpreted as reduced vagal and increased sympathetic modulation. Our resting data converge with their core observation of progressive vagal withdrawal, reflected in lower RMSSD and ln(HF) at T3, but diverge with respect to overall variability: SDNN and ln(LF/HF) remained stable across trimesters in our cohort. This discrepancy is plausibly attributable to methodological differences, seated versus supine posture, the paced rather than spontaneous breathing of several analysed segments, and the spectral estimation approach, each of which substantially influences SDNN and the LF/HF ratio. Importantly, Garg et al. characterised autonomic regulation under a single resting condition and did not probe task-related reactivity. In parallel, our group’s earlier work (Moertl et al., 2013) showed that phase synchronization between hemodynamic variables decreases at rest and after deep breathing across gestation, indicating that the cardiovascular and respiratory systems become progressively independent as pregnancy advances. The present study builds directly on both lines of evidence. Blunted parasympathetic responses to paced breathing have been described in late pregnancy (Lackner et al., 2019), but the temporal evolution and phase-specific patterns of these adaptations are still not well defined.

Accordingly, the incremental contribution of this work is not the observation that autonomic regulation changes during pregnancy, which is well established, but the integration - within a single trimester-specific, repeated-measures design - of HRV, beat-to-beat blood pressure, and cardio-respiratory phase synchronization assessed across rest, paced breathing, and a standardized cognitive challenge. This multi-phase framework shifts the focus from baseline autonomic tone to task-related autonomic reactivity, allowing the magnitude and direction of parasympathetic and sympathetic responses to be quantified at each gestational stage. It is this reactivity dimension - the progressive blunting of vagal recruitment during paced breathing and of sympathetic mobilization during cognitive load, alongside reduced vagal-driven coupling - that distinguishes our findings from prior resting-state characterisations and defines the novel physiological signal reported here.

Heart rate and blood pressure data confirmed that both sympathetic and parasympathetic branches remain functionally responsive throughout gestation: the cognitive task elicited reproducible increases in heart rate and blood pressure in all trimesters, while paced breathing consistently induced their reduction. These phase-dependent shifts indicate preserved autonomic directionality; however, the magnitude of these responses diminished in late pregnancy, suggesting a progressive reduction in autonomic dynamic range. HRV patterns clarify the nature of this adjustment. RMSSD was significantly lower at T3 compared to T1 and T2, demonstrating a reduction in beat-to-beat vagal modulation. In contrast, SDNN remained stable across gestation, indicating that overall HRV, dominated by slower oscillatory components linked to baroreflex and vascular regulation, was preserved despite vagal withdrawal. Consistently, ln(LF/HF) did not exhibit a significant main effect of trimester, reflecting parallel reductions in LF and HF power and resulting in a stable sympathovagal ratio. However, the wide interindividual variability observed, reflected by large standard deviations, points to considerable differences in autonomic adaptability among participants, likely influenced by physiological, behavioural, or psychological factors. This has been confirmed by previous exploratory studies (Brown et al., 2021). Because LF and HF power declined in parallel, the stable ratio reflects a global down-scaling of vagally linked spectral power rather than a shift toward sympathetic predominance; given that ln(LF/HF) is an indirect and debated index of sympathovagal balance (Goldberger, 1999; Billman, 2013), we anchor our interpretation on RMSSD and on ln(HF) obtained under controlled paced breathing, treating ln(LF/HF) as a supporting measure only.

At the level of reactivity, significant Time×Phase interactions across HRV indices reveal that both sympathetic and parasympathetic responses become progressively blunted in late gestation. During paced breathing, the vagal-driven increase in RMSSD and reduction in ln(LF/HF) were less pronounced in T3, indicating attenuated capacity for parasympathetic recruitment. Similarly, although the mental challenge reliably increased ln(LF/HF) and heart rate in all trimesters, the sympathetic-oriented shift was smaller in T3. This physiological attenuation parallels the slower behavioural performance observed during the cognitive task in T3, suggesting that diminished autonomic mobilization may impact task-related efficiency. These alterations in autonomic reactivity may be relevant in light of evidence that pregnancy is accompanied by structural brain changes, including reductions in gray matter volume in regions involved in cognitive and autonomic regulation (Hoekzema et al., 2017). Moreover, HRV has been linked to gray matter integrity within the central autonomic network in non-pregnant populations (Wei et al., 2018), suggesting that the blunted autonomic responses and slower cognitive performance observed in late gestation may reflect broader, pregnancy-related adaptations within neurocardiac regulatory circuits.

Phase-synchronization analyses provided complementary evidence of reduced autonomic flexibility. As expected, paced breathing elicited the strongest synchronization across RR–Resp, SBP–Resp, and SBP–RR coupling, reflecting vagally mediated alignment of cardiac, respiratory, and baroreflex rhythms. However, these synchronization peaks were consistently lower in T3, indicating that late pregnancy is characterized not only by reduced vagal tone but also by reduced vagal-driven coherence across oscillatory systems. In contrast, synchronization remained uniformly low during the cognitive task, consistent with sympathetic dominance and irregular breathing patterns; trimester differences thus emerged specifically in vagal-dependent phases, aligning with the HRV-based evidence of attenuated parasympathetic responsiveness.

Taken together, these results depict an autonomic phenotype in healthy pregnancy characterized by preserved function but reduced flexibility. Notably, the blood-pressure response to the protocol phases did not differ across trimesters (no Time × Phase interaction), indicating that short-term hemodynamic output was preserved, whereas the attenuation was specific to the autonomic domain - smaller vagal and sympathetic HRV reactivity and reduced cardio-respiratory coupling at T3 - so that homeostatic output is protected while the modulatory range that fine-tunes it is progressively narrowed. With advancing gestation, parasympathetic modulation declines, sympathetic and vagal reactivity become less pronounced, and the integration of cardiac, respiratory, and vascular rhythms shows diminished coherence. This narrowing of autonomic dynamic range likely reflects a normal physiological adaptation to the hemodynamic and metabolic demands of late pregnancy. However, these findings also delineate potential pathways through which deviations from this pattern could contribute to maladaptive trajectories in cardiometabolic diseases of pregnancy, where early loss of autonomic integration and reduced regulatory reserve may predispose to adverse maternal–fetal outcomes. Furthermore, the combined use of HRV parameters and phase-synchronization indices demonstrates the value of integrating multiple dimensions of autonomic assessment. While traditional cardiovascular measures captured expected gestational trends, these dynamic metrics revealed more subtle modifications in autonomic balance, reactivity, and multisystem coordination. This multidimensional approach highlights that healthy pregnancy involves not only changes in autonomic tone, but also adaptations in how cardiac, respiratory, and vascular systems interact under varying physiological demands. Such comprehensive profiling may improve our ability to identify early deviations from normative trajectories and to characterize autonomic dysregulation. Indeed, Chaswal et al. reported reduced HRV indices in normotensive pregnant individuals relative to non-pregnant controls, but more pronounced autonomic impairment in preeclampsia (Chaswal et al., 2018). We hypothesise that integrating autonomic function assessment at rest with standardized physiological and cognitive challenges could be more sensitive to unmask subclinical dysregulation and to identify women at increased risk for pregnancy complications. Because gross blood-pressure responses were preserved here even as autonomic reactivity declined, measures obtained under standardized challenge, particularly vagal reactivity during paced breathing and vagal-dependent coupling, may be more sensitive than resting HRV to early dysregulation; we therefore hypothesise that an early loss of these responses could mark risk for preeclampsia, gestational diabetes, or fetal growth restriction, a possibility that requires prospective testing in at-risk cohorts.

From a physiopathological perspective, the observed reduction in parasympathetic modulation and blunted sympathetically mediated responses with advancing gestation likely reflects adaptive changes in baroreflex control and neurovascular signalling. Elevated plasma volume and reduced peripheral vascular resistance necessitate increased sympathetic drive to maintain arterial pressure, but concomitant hormonal modulation (e.g., rising progesterone, oestrogen and relaxin) dampens vascular tone and resets baroreflex set points. Consequently, the baroreflex gain for sympathetic outflow is reduced while cardiovagal gain is maintained, which aligns with our finding of stable SDNN but decreased RMSSD. The decline in phase synchronisation between cardiac, respiratory and vascular oscillations during paced breathing suggests attenuated vagal−mediated coupling.

Strengths of our study include its longitudinal design, allowing within-subject comparisons across all three trimesters, and the use of a standardized measurement protocol including rest, paced brathing, and cognitive stress. The use of continuous, high-resolution physiological monitoring and validated artifact-correction algorithms further enhances data quality and interpretability. However, this study is not without limitations. Our sample consisted of only healthy pregnant women, limiting the generalizability of findings to high-risk pregnancies. The absence of postpartum measurements also restricts insight into whether observed changes revert after delivery. Additionally, while HRV is a widely accepted proxy for autonomic activity, it is influenced by multiple physiological factors and may not provide a direct measure of sympathetic nerve traffic or vagal tone. The use of ln(LF/HF) as a sympathetic-parasympathetic balance index is common but still debated in terms of specificity (Billman, 2013). The absence of a non-pregnant control group also limits our ability to separate pregnancy-specific changes from general time-related or repeated-testing effects, although our ongoing work on this protocol indicates expected time-of-day effects but no significant repeated-testing effects. Finally, several behavioral and environmental factors known to influence HRV, including sleep, physical activity, and perceived stress, were not formally recorded and remain potential unmeasured confounders, even though caffeine and food intake were standardized and the within-subject design uses each woman as her own control. In addition, respiration depth was not recorded, which limits our ability to interpret the full extent of respiratory–cardiac coupling changes.

## 5 Conclusion

Pregnancy is accompanied by both altered baseline autonomic function and a gradual reduction in the magnitude, but not the direction, of autonomic reactivity to physiological and cognitive challenges. Heart rate variability (HRV) and phase synchronization analyses revealed a possible decline in parasympathetic modulation during paced breathing and a reduced sympathetic response during the memory task. Across gestation, respiratory and cognitive challenges continued to elicit responses in the expected direction, indicating that autonomic responsiveness is preserved; the magnitude of these responses, however, was attenuated in the third trimester, consistent with a narrowing of autonomic dynamic range rather than a loss of function. Together, these findings suggest an adaptive recalibration of maternal autonomic regulation throughout gestation, promoting cardiovascular stability and fetal homeostasis at the expense of autonomic flexibility. From a clinical perspective, defining the normative trajectory of autonomic adaptation may help identify deviations associated with cardiometabolic complications of pregnancy and improve early recognition of women at risk for adverse outcomes.

## Data Availability

The raw data supporting the conclusions of this article will be made available by the authors, without undue reservation.

## Supplementary material

Supplementary Table 1 Proportion of valid heart-rate data across protocol phases and gestational time points.
