# Supplementary material for: Attenuation of short-term autonomic cardiovascular responses with advancing gestational age
Source: Front Physiol. 2026 Jul 29;17:1841598. doi: 10.3389/fphys.2026.1841598 (PMC13461391; doi:10.3389/fphys.2026.1841598)
Supplement: Supplementary file 1 [file Table1.docx]

**Supplementary Table 1. Proportion of valid heart-rate data across protocol phases and gestational time points.**

|  | R_1_ | MT_L_ | PB | MT_S_ | R_2_ | *F*-Statistics |
| --- | --- | --- | --- | --- | --- | --- |
| Valid HR (%) |  |  |  |  |  |  |
| T1 | 99.9 ± 0.5 | 99.5 ± 1.9 | 99.5 ± 1.2 | 99.5 ± 1.1 | 99.9 ± 0.4 | *Time*: F(2,132) = 0.6; p = 0.525; η_p_^2^=.01 |
| T2 | 99.7 ± 1.5 | 99.8 ± 0.8 | 99.5 ± 1.1 | 99.9 ± 0.3 | 99.8 ± 0.7 | *Phase*: F(4,264) = 1.9; p = 0.133; η_p_^2^=.03 |
| T3 | 99.6 ± 1.5 | 99.9 ± 0.4 | 99.7 ± 0.8 | 99.7 ± 0.7 | 99.8 ± 0.9 | *Time x Phase*: F(8,528) = 1.8; p = 0.10; η_p_^2^=.03 |

Data are mean ± SD (n = 67), expressed as the percentage of valid (artifact-screened) heart-rate data retained for analysis in each phase. T1: 12+1–16+0 weeks; T2: 21+1–25+0 weeks; T3: 31+0–35+0 weeks of gestation. F-statistics are from a 3 (Time) × 5 (Phase) repeated-measures ANOVA; partial eta squared (ηp²) is reported as the effect size. R1 = Rest period 1; MTL = Memory Task (learning); PB = Paced Breathing; MTS = Memory Task (solving); R2 = Rest period 2. No significant effect of Time, Phase, or Time × Phase was observed (all p > 0.10), indicating comparable signal quality across gestation and protocol phases.
